# Exploring the relationship between pain intensity, self-management of pain and pain self-efficacy on post-operative pain on day 5 after cesarean section: A cross-sectional study in Mie prefecture in Japan

**DOI:** 10.18332/ejm/194961

**Published:** 2024-11-25

**Authors:** Kyoko Takahashi, Yoko Asaka

**Affiliations:** 1 Department of Maternal and Child Health Nursing, Mie University Graduate School of Medicine, Tsu, Japan

**Keywords:** cesarean section, post-operative pain, pain management, pain self-efficacy

## Abstract

**INTRODUCTION:**

Pain self-management among women who begin child-rearing after a cesarean section (CS) has not been ascertained. This study aimed to explore the relationship among pain intensity, self-management, and self-efficacy on post-operative day (POD) 5 after CS in postpartum women.

**METHODS:**

A cross-sectional online survey was conducted on POD5 after CS. Participants were recruited through convenience sampling in Mie Prefecture, Japan, from August 2023 to April 2024. Pain was assessed using a numerical rating scale (NRS), both during activity and at rest, with higher scores indicating greater pain. Pain self-efficacy was assessed using the Japanese version of the Pain Self-Efficacy Questionnaire (PSEQ-J), and pain self-management was investigated. SPSS version 29.0 was used for data analysis, and p<0.05 was set as the significance level.

**RESULTS:**

Data from 124 participants (valid response rate: 73.8%) were analyzed. The median (IQR) of the NRS was 4.5 (3–6) during activity and 2.0 (1–4) at rest. There were significant differences according to method of oral analgesia during activity (p<0.049) and at rest (p<0.015). Multiple regression analysis revealed that NRS scores at maximum pain after CS significantly influenced pain on POD5. However, the number of oral analgesics and PSEQ-J scores were not influenced (during activity: R^2^=0.21, p<0.001, at rest: R^2^=0.12, p<0.001).

**CONCLUSIONS:**

Pain intensity during activity on POD5 was moderate and required pain-management. Acute post-operative pain-management was related to pain intensity on POD5, suggesting the importance of early post-operative pain control. Future studies are needed to examine the association between pain self-efficacy and other psychological factors.

## INTRODUCTION

Cesarean section (CS) rates have increased worldwide since the 1990s. The data for 2010–2018 from 154 countries showed that 21.1% of women gave birth by CS, and the global CS rate is estimated to be 28.5% in 2030^1^. Moreover, the CS rate in Japan increased from 19.6% in 2011 to 21.6% in 2020^2^. Thus, CS is a significant procedure in perinatal medicine. CS differs from other surgeries in that postpartum women are required to raise their newborn while experiencing pain after surgery^3^. Reportedly, CS increases the risk of postpartum depression^4^, and the association between post-cesarean pain and postpartum depression^3,5^, the importance of maternal recovery and pain management is growing in significance soon after surgery.

Wound healing relieves acute post-operative pain. Approximately 40% of patients experience severe acute post-operative pain^6^. Regarding the intensity of post-operative pain on CS, 38.5% of women experience moderate or more severe pain on post-operative day 3 (POD3)^5^ and 37–39% within three weeks^7,8^. Previous studies showed that 81.4% of women reported pain relief by three months after surgery, but only 55% reported pain relief by one month after surgery^9^. Therefore, it is estimated that approximately 30–50% of postpartum women require pain control within the first month after surgery. Recently, chronic pain, which continues for at least three months after surgery^10^, has been increasingly studied^7,8,11^. Previous studies have shown that post-operative pain after CS, progresses to chronic pain. The incidence rate has been reported to be 6–55%^10^. Some studies have shown that chronic pain is associated with the intensity of acute pain^3,7-9,12,13^, while others have shown no association^14^. Furthermore, longitudinal changes in pain after the acute stage have not been clarified.

Currently, perioperative pain management is dominated by multimodal methods that combine analgesics with different pharmacological mechanisms of action, such as opioids, nonsteroidal analgesics (NSAIDs), and acetaminophen^15,16^. However, they are mainly used for pain management during the acute post-operative phase. Studies on pain management after an acute operative period, mainly with oral medications, are limited. In general, oral analgesics are prescribed at discharge from the hospital within a week or during health checkups within a month after delivery, according to a woman’s complaints of pain in Japan. Nevertheless, information regarding the details of the prescription is scant. Furthermore, a study on pain relief methods during labor demonstrated that Japanese women’s cultural perspectives and passive attitudes influenced their decision-making processes regarding pain relief^17^. Thus, postpartum women may be reluctant to receive pain relief even after CS.

It is important to enhance the care that allows self-management of post-operative pain to be able to acquire the role of maternal role while maintaining mental health after CS. Therefore, we focused on pain self-efficacy, which has been used in chronic pain research. Pain self-efficacy is the positive belief about pain, particularly chronic pain, and confidence in one’s ability to engage successfully in activities despite experiencing pain^18^. Pain self-efficacy has been investigated increasingly in the field of chronic pain study^19,20^. Thus, the psychological response to pain or the ability to control pain in daily life after starting child-rearing is a necessary perspective for evaluating post-operative pain control. Once the acute stage has passed, being able to control pain by taking analgesics means feeling able to carry out daily activities including childcare, which means that pain self-efficacy is high. Nonetheless, to our knowledge, no studies have examined the relationship among post-operative pain intensity, pain management, and pain self-efficacy in postpartum women after CS.

In summary, it is necessary to clarify the longitudinal changes in pain after the acute stage, pain management, and pain self-efficacy through a longitudinal study from within a week to one month, considering the timing of discharge from the hospital and health checkups in Japan. Clarifying post-operative pain, pain control, and self-efficacy after the acute stage will improve support for postpartum women after CS to maintain their mental health and to acquire the role of mother.

This study analyzes post-operative pain on POD5 after surgery, as a part of the longitudinal study. As previous studies have shown that numerous women have post-operative pain on POD5 before discharge from the hospital^5,7,8^, a survey at this point can be used to consider support for pain self-management during hospitalization. This study explores the relationship among pain intensity, self-management, and self-efficacy on POD5 after CS in postpartum women.

## METHODS

### Study design and setting

This cross-sectional study was part of a longitudinal study conducted up to one month after delivery. This article presents cross-sectional data on the fifth day after CS. Convenience sampling was conducted to select the delivery facilities in Mie Prefecture, Japan. In Japan, 70% of CS procedures are performed in hospitals and 30% in clinics^2^. Based on this evidence, delivery facilities that cooperated with this study were selected through the following process: all five hospitals certified as perinatal medical centers and clinics that handled at least 300 deliveries per year were selected as candidates. Of the 35 delivery facilities in the prefecture, we obtained research cooperation from seven facilities: two comprehensive perinatal medical centers, two regional perinatal medical centers, and three clinics. The total number of annual births in 2021 at these seven facilities accounted for 40%.

### Participants

The inclusion criteria were as follows: 1) CS after 32 weeks of singleton pregnancy; 2) both women and newborns were healthy at the time of participation in this study; 3) aged ≥18 years; 4) having a Japanese-speaking background; and 5) consent to participate. The exclusion criteria were as follows: 1) had undergone total hysterectomy during CS; 2) had been diagnosed with a mental illness or serious comorbidities; and 3) withdrawal of consent.

The sample size was calculated with a response rate of 0.5, a sampling error of 10 percentage points, and a confidence level of 95% (λ=1.96). Based on these calculations, the required number of participants was estimated to be 96. As this was a longitudinal survey, the response rate was assumed to be 50%. Therefore, the number of distributions was set to 200.

### Ethical considerations

Ethical considerations were explained in writing to the research collaborators who were informed that participation in the study was voluntary and that not agreeing to participate would not cause any disadvantages. When they answered the questionnaire, their intention to consent was confirmed, and they provided consent. The questionnaires were answered anonymously. This study was approved by the Research Ethics Committee of Mie University (No. U2023-010: Date: 19 July 2023). The researcher explained the study’s aim to the nursing managers to obtain approval before data collection. The person in charge of each collaborating institution informed the candidate participants that participation in the research was voluntary, with a document explaining the study. Candidate participants scanned the QR code using a smartphone or other device, provided consent for the online survey, and completed the survey using Google Forms.

### Data sources and variables

An online questionnaire was administered. The questionnaire comprised scales that had already been developed for pain intensity and self-efficacy and questions about pain self-management and demographic data selected by researchers. The questionnaire was pretested, and no modification of the scale was necessary. All postpartum women who met the inclusion criteria were recruited for the survey. The data collection period was from August 2023 to April 2024 on POD5, July on one month.

*Demographic data*

Demographic data of the study participants included age, parity, number of previous CS, gestational weeks of delivery, and type of CS (scheduled, emergency, or other).

*Post-operative pain intensity*

Pain intensity was measured using a numerical rating scale (NRS) ranging from 0 to 10 (0: no pain; 1–3: mild pain; 4–6: moderate pain; 7–10: severe pain). This scale is widely used in post-operative pain management^21^. In this study, participants reported average pain in the past 24 hours, both during activity and at rest. Additionally, the participants were asked about the number of days after surgery when the pain was maximum and the NRS score at that time. All relevant pain types were selected from the following options: wound pain, afterpains, and visceral, intestinal, or nerve pain. Pain during activity was recorded as yes or no, while holding the baby, breastfeeding, changing the baby’s diaper, or bathing the baby (Supplementary file Material 1).

*Pain self-efficacy questionnaire*

The Pain Self-Efficacy Questionnaire (PSEQ) measures self-efficacy and assesses confidence in performing movement during pain^18^. Ten items were rated on a 6-point Likert scale ranging from 0 (not at all confident) to 6 (completely confident), with higher scores indicating higher self-efficacy in performing movements. Moreover, a score of ≥40 is considered to indicate high pain self-efficacy. This study used the Japanese version of PSEQ (PSEQ-J)^22^. Although this scale was originally developed to measure self-efficacy in chronic pain, it has recently been used to measure postoperative acute pain^23^. Participants were asked to answer the scale while imagining their lives after discharge from the hospital (Supplementary file Material 2). We obtained permission from the developer of the scale to use it for post-operative pain. Cronbach’s alpha coefficients for the PSEQ and PSEQ-J were 0.92^18^ and 0.94^22^, respectively. In this study, the alpha coefficient was 0.94.

*Self-management of pain*

The participants were asked about their post-operative pain control methods on POD5 (Supplementary Material 3). Oral antipyretic analgesics were administered to the participants, and the method of oral analgesia was selected from a fixed time, preventive, on-demand, or other. The number of oral analgesics taken per day was selected as once a day, twice a day, three times a day, four or more times a day, or none.

### Variables

In this study, the objective variables were NRS scores during activity and at rest, and the explanatory variables were the maximum post-operative NRS score, method of oral administration, number of oral analgesics taken per day, and PSEQ-J. The confounding factors were gestational age, age, parity, history of CS, and type of CS based on previous studies.

### Statistical analysis

The Shapiro-Wilk test was performed to determine normality. As the NRS scores were not normally distributed, nonparametric tests were used. Spearman’s rank correlation coefficient was calculated between the NRS scores, maximum pain intensity, and the PSEQ-J score. The Mann-Whitney U test or Kruskal-Wallis test was used to examine the relationship between the NRS score, pain management, and demographic variables on POD5. For the Kruskal-Wallis test, if significant differences were found, Bonferroni’s multiple comparison test was performed to test for all differences between levels. Lastly, multiple regression analysis was performed using the forced entry method with NRS scores at rest and during activity on POD5. As no significant difference was confirmed by the nonparametric tests, the demographic variables set as confounding factors were not included in the multiple regression analysis. The questionnaires with missing data were excluded from the analysis. IBM SPSS (version 29.0; SPSS Inc., Chicago, IL, USA) was used for data analysis, and statistical significance was set at p<0.05.

## RESULTS

The survey forms were distributed to 218 participants, and 168 were returned (response rate: 77.1%). After excluding those with missing data, 124 responses were analyzed as the study subjects (validity response rate: 73.8%).

The participants were aged 25–45 years (mean = 34.1 ± 4.53), and 44 (35.5%) were primiparas, while 80 (64.5%) were multiparas. Among multiparous women, 63 (78.8%) had a history of CS. Of these, 112 (90.3%) delivered full-term births, 81 (65.3%) underwent scheduled CS, 25 (20.2%) underwent emergency CS, and others 18 (14.5%) (data not shown).

The median (interquartile range, IQR) NRS score during activity was 4.5 (3–6), and that at rest was 2.0 (1–4) on POD5. Furthermore, 45 (36.3%) postpartum women reported NRS scores of <4 during activity and 92 (74.2%) at rest (Table 1). Among those with pain, wound pain was present in 116 (93.5%), afterpains in 103 (83.1%), visceral pain in 97 (78.2%), intestinal pain in 28 (22.6%), nerve pain in 10 (8.1%), and other in 7 (5.6%). The numbers of reported pain during childcare activities were as follows: 56 (45.2%) for breastfeeding, 55 (44.4%) for changing the baby’s diaper, 45 (36.3%) for holding the baby, and 34 (27.4%) for bathing the baby (data not shown).

**Table 1:** Pain intensity assessed by numerical rating scale (NRS), 5 days after cesarean section, a cross-sectional study in Mie prefecture in Japan

*NRS*	*During activity*		*At rest*	
	*n*	*%*	*n*	*%*
0	0	0	24	19.4
1	10	8.1	31	25.0
2	18	14.5	17	13.7
3	17	13.7	20	16.1
4	17	13.7	12	9.7
5	17	13.7	7	5.6
6	15	12.1	3	2.4
7	9	7.3	5	4.0
8	9	7.3	2	1.6
9	5	4.0	1	0.8
10	7	5.6	2	1.6
Median (IQR)	4.5 (3–6)		2.0 (1–4)	

NRS is a widely used tool for assessing pain intensity, it is rated on an 11-point scale from 0 to 10, with higher scores indicating greater pain intensity. IQR: interquartile range.

The maximum post-operative pain was felt by 75 women (60.5%) on POD 1, 33 (26.6%) women on POD 0, 14 women (11.3 %) on POD 2, and 2 women (1.6%) after POD 2. The median (IQR) NRS score for maximum pain intensity was 8.0 (7–9). Spearman’s rank correlation coefficients between the NRS scores of the maximum pain intensity after surgery and the NRS scores on POD5 were calculated. The results showed a significant moderate correlation during activity (rs=0.42, p<0.01) and at rest (rs=0.30, p<0.01). The Mann-Whitney U and Kruskal-Wallis tests showed no significant differences between the NRS scores during activity and at rest and the demographic variables (Supplementary file Table 1).

Regarding pain control on POD5, 112 (90.3%) participants received oral medication (Table 2). Two nonsteroidal anti-inflammatory drugs (NSAIDs) – Loxoprofen sodium hydrate and diclofenac sodium – and an acetaminophen antipyretic analgesic were administered. The most common response was preventive medications (n=49; 39.5%). Additionally, the most common response to the number of oral analgesics administered per day was three times per day (n=41; 33.1%).

**Table 2:** Use of oral analgesia 5 days after cesarean section, a cross-sectional study in Mie prefecture in Japan

	*n*	*%*
**The number of oral analgesia per day**		
1	13	10.5
2	30	24.2
3	41	33.1
≥4	28	22.6
0	12	9.7
**Method**		
Fixed time	26	21.0
On-demand	37	29.8
Preventive	49	39.5
Other	3	2.4
**Refrain from taking oral analgesia**		
Yes	49	39.5
No	75	60.5
**Antipyretic analgesics^a^**		
Loxoprofen sodium hydrate (NSAIDs)	97	78.2
Acetaminophen	57	46.0
Diclofenac sodium (NSAIDs)	9	7.3
Other	2	1.6

Participants were asked to choose one option that applied to them regarding the number of times, the method of administration, and whether or not they had ever had to refrain from medication.

a Data were obtained from multiple choices. NSAIDs: non-steroidal anti-inflammatory drugs.

**Figure 1:**
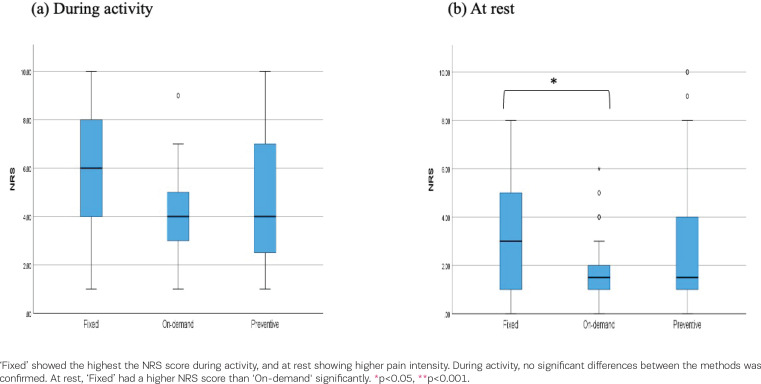
The association between the numerical rating scale score (NRS) and method of oral analgesia; a) numerical rating scale (NRS) score during activity, and b) at rest, on the fifth day after cesarean section by the method of oral medication

**Figure 2:**
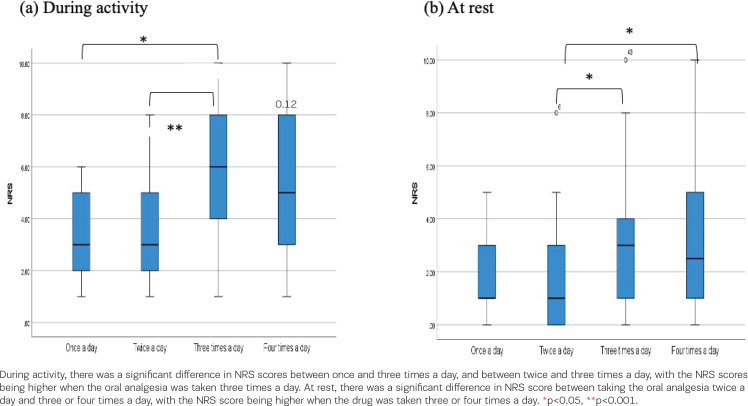
The association between the numerical rating scale score (NRS) and the number of oral analgesia; a) numerical rating scale (NRS) score during activity, and b) at rest, on the fifth day after cesarean section according to the number of oral medications taken per day

The NRS scores on POD5 by method and number of oral analgesics were compared using the Kruskal-Wallis test. The subjects of this analysis were 112 participants who took oral medication. There were significant differences according to method during activity (z=6.045, p=0.049) and at rest (z=8.347, p=0.015). Bonferroni’s multiple comparison test revealed no significant differences between the methods during the activity. The NRS score of ‘fixed time’ was significantly higher than ‘on-demand’ (p=0.012) at rest (Figure 1). Moreover, there were significant differences in the NRS scores according to the number of oral analgesics administered during activity (z=17.104, p<0.001) and at rest (z=10.653, p=0.014). Bonferroni’s multiple comparison test revealed significant differences between the NRS scores of once and three times taken a day (p=0.022) and twice and three times taken a day (p=0.004) during activity. Participants taking oral analgesia three times per day had significantly higher NRS scores than those taking oral analgesia once or twice per day during activity. The participants taking oral analgesia three times and four times taken per day had significantly higher NRS scores than those taking oral analgesia twice per day taken at rest (three times-twice, p=0.036: four times-twice, p=0.049) (Figure 2).

Spearman’s rank correlation coefficient was calculated to examine the correlation between NRS and PSEQ-J scores on POD5. The results showed no significant correlations (during activity: rs= -0.128, p=0.155; at rest: rs= -0.169, p=0.061). The median (IQR) of the PSEQ-J score was 29 (18–41). The Kruskal-Wallis test was used to analyze the PSEQ-J on POD5 according to the method and number of oral analgesics, and no significant differences were observed in the PSEQ-J, method (z=1.76, p=0.63), or number (z=5.83, p=0.15).

A multiple regression analysis was performed with NRS scores on POD5 during activity and at rest as the dependent variable and NRS scores at maximum pain intensity, number of oral analgesics, and PSEQ-J score as the independent variables (Table 3). The results showed that the NRS score at maximum pain significantly influenced pain on POD5 and the number of oral analgesics and PSEQ-J score were not associated with NRS scores both during activity (at maximum pain intensity: β=0.424; B=0.796; 95% CI: 0.496–1.096, p<0.001,the number of oral analgesics: β=0.150; B=0.355; 95% CI: 0.020–0.690, p<0.064, PSEQ-J score: β= -0.108; B= -0.019; 95% CI: -0.047–0.009, p<0.184), and at rest (at maximum pain intensity: β=0.291; B=0.498; 95% CI: 0.210–0.787, p<0.001,the number of oral analgesics: β=0.129; B=0.262; 95% CI: -0.079–0.604, p<0.131, PSEQ-J score: β= -0.164; B= -0.026; 95% CI: -0.053–0.001, p<0.057). The adjusted R^2^ was 0.21 (p<0.001) during activity, and 0.12 (p<0.001) at rest.

**Table 3:** Multiple linear regression analysis of influencing factors associated with NRS score, 5 days after cesarean section, a cross-sectional study in Mie prefecture in Japan (N=124)

*Independent variable*	*B (95% CI)*	*SE*	*β*	*t*	*p*	*VIF*
**During activity^a^**						
Maximum pain intensity within POD5	0.796 (0.496–1.096)	0.152	0.424	5.251	<0.001**	1.012
The number of oral analgesia per day	0.355 (0.020–0.690)	0.179	0.150	1.870	0.064	1.004
PSEQ-J	-0.019 (-0.047–0.009)	0.014	-0.108	-1.335	0.184	1.009
**At rest^b^**						
Maximum pain intensity within POD5	0.498 (0.210-0.787)	0.146	0.291	3.418	<0.001**	1.012
The number of oral analgesia per day	0.262 (-0.079–0.604)	0.172	0.129	1.521	0.131	1.004
PSEQ-J	-0.026 (-0.053–0.001)	0.014	-0.164	-1.923	0.057	1.009

a F=11.867. p<0.001. R^2^=0.229. Adjusted R^2^=0.210. The dependent variable was the NRS score during activity.

b F=6.534. p<0.001. R^2^=0.140. Adjusted R^2^=0.119. The dependent variable was the NRS score at rest. B: partial regression coefficient. β: standardized partial regression coefficient. NRS is a widely used tool for assessing pain intensity, it is rated on an 11-point scale from 0 to 10, with higher scores indicating greater pain intensity. The NRS scores both during activity and at rest were influenced by the NRS score at the time of maximum post-operative pain. A higher NRS score at the time of maximum post-operative pain was a factor in increasing the NRS on the fifth post-operative day. POD: post-operative day. PSEQ-J: Pain Self-Efficacy Questionnaire-Japan.

* p<0.005.

** p<0.001.

## DISCUSSION

This is a novel study that focuses on post-operative pain, pain management, and pain self-efficacy on POD5 after CS. The results of the study highlight the need for pain self-management in postpartum women after the acute post-operative period.

On POD5, the NRS score indicated moderate pain intensity during activity and mild pain intensity at rest. Approximately 80% of the postpartum women experienced ‘afterpains’ and ‘visceral pain’ in addition to the wound pain. Thus, the pain reflected in the NRS included wound pain and multiple other types of pain. Furthermore, pain was experienced during the childcare movement. Optimizing post-operative analgesia has been emphasized as important. The British College of Anesthetists recommended that 90% of patients should be satisfied with their pain management, with a pain score of less than 4^24^. The percentage of participants whose NRS score of less than 4 was 36.3% during activity in this study. Thus, the score in this study indicates that pain control after CS on POD5 must be improved, particularly during activity.

Multiple regression analysis revealed that the NRS scores at maximum pain after CS significantly influenced pain on POD5, and the NRS score at maximum pain after CS explained 21% of the pain during activity and 12% of pain at rest, respectively. As the explanatory rate is low, other variables may explain the NRS scores on POD5. Nevertheless, this result suggests that reducing the maximum post-operative pain may lead to a reduction in POD5 pain. This result supports previous studies, which reported the association of the intensity of acute pain and chronic pain^3,7-9,12,13^. Thus, pain management during the acute post-operative period is crucial for predicting pain outcomes later in the recovery period, particularly during activity.

The results of the method and frequency of oral analgesic intake showed that the NRS was the highest for fixed-time administration at rest, and the NRS score was the highest for those taking the medication three times a day during activity and at rest. Thus, the use of oral medications should be considered to reduce pain intensity. Fixed time involves administration at a predetermined time regardless of the intensity of pain^25^ and was prescribed as an aftermeal medication in this study. In an evaluation of the analgesic effects of fixed-time interval and on-demand, administration at the time the patient requests pain medication and post-operative oral analgesics, a previous study reported that a fixed-time interval is more effective than on-demand^25^. However, this study was conducted on acute pain after surgery and used medications with high pain-relieving properties, making it difficult to compare their results with those of this study. Additionally, the preventive approach resulted in lower pain scores. Preventive analgesia suppresses the production of pain-causing substances and reduces post-operative pain by blocking the nerves that transmit pain stimuli before pain occurs or preventing pain stimuli from reaching the central nervous system. Reportedly, this method has a greater analgesic effect^26^. However, in this study, no significant difference in pain intensity was found between on-demand and preventive in both during activity and at rest. Furthermore, the number of times of oral administration is considered to be related to the method of oral administration. Thus, further consideration of the optimal method of oral analgesia administration is required.

This study measured pain self-efficacy using the PSEQ-J in postpartum women with CS. Compared to previous studies, the results of this study showed low pain self-efficacy. In previous studies of patients from the general surgery, gynecology, and thoracic departments, the mean PSEQ score exceeded 50 points^23,27^. This reflects the low number of participants scoring less than 4 in pain assessments using the NRS, particularly during the activity in this study. Furthermore, unlike other surgeries, postpartum women begin childcare and engage in activities related to caring for newborns after CS. Although more than 60% of the participants in this study were multiparous, POD5 was the time to learn about the characteristics of the newborns and acquire appropriate care techniques^28^. Therefore, being unaccustomed to life with a newborn may have influenced their responses to the PSEQ-J.

The results of the multiple regression analysis showed that self-efficacy had no effect on NRS scores on POD5. However, previous studies have reported an association between the PSEQ and post-operative pain intensity^27,29^. As shown by the relationship between the number and method of oral analgesia in this study, the participants included those who did not receive effective analgesics. Pain self-efficacy is achieved through psychological reactions to pain management strategies. This could explain why no such association was observed.

### Strengths and limitations

This study addresses post-operative pain self-management and self-efficacy in the post-operative acute phase. Although this study was conducted in one prefecture in Japan, the ages of the participants in this study correspond to the general Japanese childbirth population, which is 30–35 years in Japan^30^. Moreover, as the inclusion and exclusion criteria were set for this study, participants where neither the mother nor the child was in a critical condition were selected. Furthermore, as the participants were asked about their pain in the past 24 hours, recall bias was minimal. However, this study had some limitations. First, medical information such as the intraoperative anesthesia method and CS technique was not collected from the medical records. As this information is considered a factor that influences post-operative pain intensity^13,31-33^, future analyses that consider analgesic methods from the intraoperative period to the acute phase will be necessary. Second, this was a cross-sectional study; thus, we only provided associations between variables rather than causal relationships. Third, a previous study reported that the pain self-efficacy is influenced by the assessment of physically felt pain, as indicated by the NRS, and other psychological factors such as pain sensitivity and catastrophizing^27^. Future studies are necessary to examine the association between pain self-efficacy and the psychological factors that contribute to improved pain control after CS.

## CONCLUSIONS

Our survey showed that pain intensity during activity on POD5 was classified as moderate, and only 36.3% reported an NRS score of <4 during activity. Moreover, the factor that influenced pain on POD5 was maximum pain intensity after CS. These results suggest that reducing the maximum post-operative pain may lead to reducing POD5 pain and that oral medication should be considered to reduce pain intensity. No significant relationship was found between pain self-efficacy and NRS score. Further studies are necessary to examine the association between pain self-efficacy and other psychological factors.

## Supplementary Material



## Data Availability

The data supporting the findings of this study are available from the first author upon request.
