# Supplementary material for: Exploring the relationship between pain intensity, self-management of pain and pain self-efficacy on post-operative pain on day 5 after cesarean section: A cross-sectional study in Mie prefecture in Japan
Source: Eur J Midwifery. 2024 Nov 25;8:10.18332/ejm/194961. doi: 10.18332/ejm/194961 (PMC11587602; doi:10.18332/ejm/194961)
Supplement: Supplementary file 1 [file EJM-8-69-s1.pdf]

Supplemental Material 1.

Questionnaire about postoperative pain

1. Please tell us about the most intense pain you have ever felt after a Cesarean section.

1) When did you feel the most pain after your Cesarean section?

1. Day of surgery
2. 1st day after surgery
3. 2nd day after surgery
4. 3rd day after surgery
5. 4th day after surgery
6. 5th day after surgery

2） How intense was the pain you felt the most after your Caesarean section?
Please choose the number (0-10) that best describes your pain and check one.

A "0" represents no pain at all and a "10" represents the worst pain you can imagine.

0 1 2 3 4 5 6 7 8 9 10

3) Regarding pain after your Cesarean section, please tell us about the pain you have felt in the past 24 hours.
Please check the number (0-10) that most closely applies.
"0" represents no pain at all, and "10" represents the worst pain you can imagine.

Average pain during activity

0 1 2 3 4 5 6 7 8 9 10

Average pain at rest

0 1 2 3 4 5 6 7 8 9 10

2. Please choose the most appropriate answer for the pain you have experienced in the past 24 hours.

1） Please choose all the movements that cause you pain. If there are none, choose "None."
If the movement is not listed, please write "Other."

1. When breastfeeding
2. When holding the baby
3. When changing a diaper
4. When bathing
5. No painful movements
6. Other

2) Regarding the pain you felt, please choose all that apply (multiple answers allowed). If you do not know what type of pain it is, please choose "I don't know." If you do not have pain, please choose "None." If you have a pain that is not listed in the categories, please write it in "Other."

1. Pain at the wound or on the surface
2. Internal pain or pulling pain
3. Afterpains (pain caused by uterine contractions)
*Afterpains refer to pain similar to menstrual cramps caused by uterine contractions during or after breastfeeding/expressing milk, or after using uterine contraction medication.
4. Pain that feels like your intestines moving
5. Pins and needles
6. Don't know
7. None
8. Other

Supplemental Material 2.

Questionnaire about painself-efficacy

Please tell us your current level of confidence that you can do these things even though it is painful. 0 being "Not at all confident " and 6 being "completely confident."
This questionnaire does not ask whether you have actually done the following things in the past, but rather asks "Are you confident that you can do these things at this time, even though it is painful?"

1. I can enjoy things, despite the pain.

Not at all confident Completely confident
0 6

2. I can do most of the household chores (e.g. tidying-up, washing dishes, etc.), despite the pain.

Not at all confident Completely confident
0 6

3. I can socialize with my friends or family members as often as I used to do, despite the pain.

Not at all confident Completely confident
0 6

4. I can cope with my pain in most situations.

Not at all confident | Completely confident
0 | 6

5. I can do some form of work, despite the pain. ('work' includes housework, paid and unpaid work).

Not at all confident | Completely confident
0 | 6

6. I can still do many of the things I enjoy doing, such as hobbies or leisure activity, despite pain.

Not at all confident | Completely confident
0 | 6

7. I can cope with my pain without medication.

Not at all confident | Completely confident
0 | 6

8. I can still accomplish most of my goals in life, despite the pain.

Not at all confident Completely confident

0 6

9. I can live a normal lifestyle, despite the pain.

Not at all confident Completely confident

0 6

10. I can gradually become more active, despite the pain.

Not at all confident Completely confident

0 6

Supplemental Material 3.

Questionnaire about pain-self management

1. Please answer regarding the use of painkillers after a Cesarean section.

1) Please answer the following questions about the use of painkillers for pain after a Cesarean section within the past 24 hours. *Excluding use for reasons other than pain after a Cesarean section.

1. Once a day
2. Twice a day
3. Three times a day
4. Four or more times a day
5. Not taking medication ⇒ Go to 3) (skip one question)

2）Please tell us about the use of painkillers for pain after a Cesarean section within the past 24 hours. If none of the options apply to you, please choose "other." *This does not include cases where the painkiller was used for purposes other than pain after a Cesarean section.

1. Take regularly at set times: fixed time
2. Take only when pain is severe: on-demand
3. Take preventatively before pain becomes severe: preventive
4. Other

3）Please choose all that apply regarding the names of painkillers you have taken (in the past 24 hours) (multiple answers allowed). If there are no applicable medications, please write in "Other." If you are not using any, please choose "None."

1. Loxonin (loxoprofen): NSAIDs
2. Calonal (acetaminophen): acetaminophen
3. Voltaren (diclofenac) : NSAIDs
4. None
5. Other

Supplementary table 1.

NRS scores of the participants by demographic variables.

			During Activity			At rest		
Variables	Categories	n	median	IQR	*p*	median	IQR	*p*
age [a]	Under 34	71	5.0	3.0-6.0	0.760	2.0	1.0-4.0	0.460
	Over 35	53	4.0	2.5-6.5		2.0	1.0-3.5	
parity [b]	1	44	4.0	2.0-6.0	0.424	3.0	1.0-3.0	0.623
	2	57	5.0	2.5-6.0		2.0	1.0-4.0	
	3 or more	23	4.0	2.0-6.0		3.0	1.0-3.0	
Previous CS [a]	Yes	61	5.0	3.5-7.0	0.678	3.0	1.0-4.0	0.272
	No	63	5.0	3.5-7.0		3.0	1.0-4.0	
GA [a]	Full-term	105	4.0	3.0-6.0	0.957	2.0	1.0-3.0	0.996
	Preterm	12	5.0	1.3-7.8		1.5	0.3-4.8	
Type of CS [b]	Scheduled	81	4.0	2.0-6.0	0.212	2.0	1.0-3.0	0.208
	Schedule change	18	4.5	2.8-9.0		2.5	1.0-4.3	

	from scheduled CS							
	emergency	25	5.0	3.5-7.0		3.0	1.0-4.0	

This table shows the results of a statistical test of NRS scores based on demographic variables. There were no significant differences in NRS scores by demographic variables.
The superscript a of the variable indicates the analysis result using the Mann-Whitney-U test, and the superscript b indicates the analysis result using the Kruskal-Wallis test.

NRS:Numerical Rating Scale, which assess pain intensity 0-10 with higher scores indicating greater pain.
CS: Cesarean Section, GA:Gastational age at the time of CS,

© 2024 Takahashi K. and Asaka Y.
